# Clinical and Genetic Characteristics of 153 Chinese Patients With X-Linked Hypophosphatemia

**DOI:** 10.3389/fcell.2021.617738

**Published:** 2021-06-01

**Authors:** Xiaoyun Lin, Shanshan Li, Zhenlin Zhang, Hua Yue

**Affiliations:** Shanghai Clinical Research Center of Bone Diseases, Department of Osteoporosis and Bone Diseases, Shanghai Sixth People’s Hospital Affiliated to Shanghai Jiao Tong University, Shanghai, China

**Keywords:** X-linked hypophosphatemia, clinical features, mutational spectrum, intact fibroblast growth factor-23, genotype-phenotype correlation

## Abstract

X-linked hypophosphatemia (XLH) is caused by inactivating mutations in the *phosphate-regulating endopeptidase homolog, X-linked* (*PHEX*) gene, resulting in an excess of circulating intact fibroblast growth factor-23 (iFGF-23) and a waste of renal phosphate. In the present study, we retrospectively reviewed the clinical and molecular features of 153 Chinese patients, representing 87 familial and 66 sporadic cases with XLH. A total of 153 patients with XLH presented with signs or symptoms at a median age of 18.0 months (range, 9.0 months–26.0 years). Lower-limb deformity was the most frequent clinical manifestation, accounting for 79.1% (121/153). Biochemical screening showed increased serum levels of iFGF23 in patients with XLH, with a wide variation ranging from 14.39 to 730.70 pg/ml. Median values of serum iFGF23 in pediatric and adult patients were 94.87 pg/ml (interquartile range: 74.27–151.86 pg/ml) and 72.82 pg/ml (interquartile range: 39.42–136.00 pg/ml), respectively. Although no difference in circulating iFGF23 levels between these two groups was observed (*P* = 0.062), the proportion of patients with high levels of circulating iFGF23 (>42.2 pg/ml) was greater in the pediatric group than in the adult group (*P* = 0.026). Eighty-eight different mutations in 153 patients were identified, with 27 (30.7%) being novel. iFGF23 levels and severity of the disease did not correlate significantly with truncating and non-truncating mutations or N-terminal and C-terminal *PHEX* mutations. This study provides a comprehensive description of the clinical profiles, circulating levels of iFGF23 and gene mutation features of patients with XLH, further enriching the genotypic spectrum of the diseases. The findings show no evident correlation of circulating iFGF23 levels with the age or disease severity in patients with XLH.

## Introduction

X-linked hypophosphatemia (XLH, OMIM 307800), a rare disorder of phosphorus metabolism, is the most common heritable form of rickets, with an incidence of approximately one in 20,000 births (Carpenter, 1997). The primary physiological trait of the disease is leakage of phosphate from the kidney, which leads to hypophosphatemia and defects in bone mineralization.

XLH is caused by inactivating dominant mutations in the *phosphate-regulating endopeptidase homolog X-linked* (*PHEX*) gene. The *PHEX* gene is located on Xp22.1 and exhibits homology with a family of zinc metalloproteases, including neprilysin (NEP), the Kell antigen (KELL) and endothelin-converting enzymes 1 and 2 (ECE-1 and ECE-2) (Francis et al., 1997). *PHEX* is expressed in osteocytes and odontoblasts, and loss-of-function *PHEX* mutation results in excess circulating fibroblast growth factor 23 (FGF23), leading to hypophosphatemia (Kinoshita and Fukumoto, 2018). However, studies to date on circulating levels of FGF23 in XLH patients are contradictory. For example, several studies report elevated serum intact FGF23 (iFGF23) values in the majority of patients with XLH (Yamazaki et al., 2002; Jonsson et al., 2003; Zhang et al., 2019), whereas another study indicates serum iFGF23 values virtually in the reference range (Weber et al., 2003). Thus, we conducted a study to determine circulating iFGF23 concentrations in XLH patients.

For decades, conventional therapy (multiple daily doses of oral phosphate and calcitriol) was the only treatment option for patients with XLH; the partial efficacy of this approach has been indicated in several reports (Verge et al., 1991; Mäkitie et al., 2003a; Fuente et al., 2017). However, long-term treatment is frequently accompanied by a series of complications (Santos et al., 2013; Linglart et al., 2014). Recently, a new targeted therapy for XLH, i.e., the recombinant human monoclonal antibody against FGF-23 burosumab, has been approved in the United States and Europe and has achieved significantly greater therapeutic effect and safety in patients with XLH than conventional therapy (Imel et al., 2019). Because this new therapy will soon be available in China, a depiction of the general traits of Chinese individuals with XLH is important. Our center has previously reported several XLH cases in the Chinese population (Kang et al., 2012; Yue et al., 2014; Li et al., 2016); however, these reports do not comprehensively summarize the clinical and genetic characteristics of XLH patients because of their small sample sizes. Hence, we conducted this study by enlarging the study population to further systematically delineate the clinical and genetic spectrum of XLH and to explore circulating iFGF23 levels in these patients and their relationship with clinical and genetic parameters.

## Materials and Methods

### Subjects

This study was approved by the Ethics Committee of Shanghai Jiao Tong University Affiliated Sixth People’s Hospital. This study retrospectively reviewed the clinical and molecular features of 153 patients with XLH who were identified with *PHEX* gene mutations from 2008 to 2019. These patients included 87 familial cases and 66 sporadic cases, from 105 unrelated pedigrees. Hypophosphatemic patients with other causes (autosomal dominant hypophosphatemic rickets, autosomal recessive hypophosphatemic rickets, tumor-induced osteomalacia, hereditary hypophosphatamic rickets with hypercalciuria, etc.) were excluded in this study. One hundred and sixteen of the 153 patients were orally administered phosphate 20–40 mg/kg per day divided into five doses per day and calcitriol 0.25–0.5 μg per day; follow-up data for these patients were recorded when available.

### Clinical Features

Basic information was collected, including sex, age at diagnosis, and height converted into standard deviation scores (SDS) using standardized growth charts for Chinese children and adolescents (Li et al., 2009). Clinical rachitic signs or symptoms in these patients were recorded (bowed lower extremities, abnormal gait, short stature, dental disease, etc.). Radiography of bilateral posteroanterior wrists and knees was performed, and the Rickets Severity Score (RSS) was evaluated using Thacher’s method (Thacher et al., 2000). The severity score of rickets is scaled from 0 (normal) to 10 (severe).

### Biochemical Measurements

Relevant biochemical tests included serum phosphorus, calcium, total alkaline phosphatase (ALP), intact parathyroid hormone (PTH), 25-hydroxy-vitamin D (25OHD), β-CrossLaps of type 1 collagen containing cross-linked C-telopeptide (β-CTX), and serum osteocalcin (OC), in the form of N-terminal mid-molecule fragments and creatinine.

Serum phosphorus, calcium, and ALP levels were measured using a Hitachi 7,600-020 automatic biochemistry analyzer. Serum PTH, 25OHD, β-CTX, and OC concentrations were assessed using an automated Roche electrochemiluminescence system.

### Serum iFGF23 Measurement

Serum samples were collected to measure iFGF23 levels at each patient’s first visit to Shanghai Jiao Tong University Affiliated Sixth People’s Hospital. All samples were stored at −80°C until analysis. In total, 62 samples were available for measuring iFGF23. Serum iFGF23 levels were evaluated using a two-site ELISA kit (KAINOS Laboratories, Inc., Tokyo, Japan) with a detectable concentration range from 3 to 800 pg/ml. The reference range for serum iFGF23 is 16.1–42.2 pg/mL (Zhang et al., 2019).

### Sanger Sequencing for *PHEX* Gene Mutations

Genomic DNA was extracted from peripheral blood leukocytes using a DNA extraction kit (Lifefeng Biotech, Shanghai). The DNA sequence for the *PHEX* gene was obtained from an online database (GenBank accession NO. NC _000012.). The PCR and sequencing primers were the same as those used in our previous study and were designed using Primer 3 software^1^. All 22 exons and exon-intron boundaries of the *PHEX* gene were amplified by polymerase chain reaction (PCR). Direct sequencing was performed using BigDye Terminator Cycle Sequencing Ready Reaction Kit, version 3.1 (Applied Biosystems, Foster, CA, United States), and the product was analyzed with an automated ABI 3730 sequencer (Foster, CA, United States). Single-nucleotide polymorphisms (SNPs) were identified using Polyphred^2^ and novel mutations using HGMD.

### Multiplex Ligation-Dependent Probe Amplification Analysis

Multiplex ligation-dependent probe amplification (MLPA) analysis was performed to detect large deletion/duplication mutations in patients for whom direct DNA sequencing did not reveal *PHEX* mutations. MLPA analysis was performed according to the manufacturer’s instructions (Salsa MLPA Kit P223 *PHEX*, Version 01, MRC-Holland, Amsterdam, Netherlands), and the product was analyzed using an ABI 3730XL sequencer (Applied Biosystems, Foster City, CA, United States) and the Coffalyser software program (MRC-Holland, Amsterdam, Netherlands).

### Statistical Analyses

All data were analyzed using IBM SPSS Statistics (version 26.0; SPSS Inc., Chicago, IL, United States). The Kolmogorov–Smirnov test was employed to detect the normality of the distribution of continuous variables. Normally distributed data are presented as the mean ± SD, and between-group differences were assessed with independent-sample *t* tests. Non-normally distributed data are expressed as medians (25th and 75th percentiles), and intergroup differences were evaluated with the Mann–Whitney *U* test. Categorical variables are described as frequencies or percentages, and intergroup comparisons were analyzed with Fisher’s exact test. Correlations between continuous variables were analyzed with the Spearman rank correlation coefficient. A two-tailed value of *P* < 0.05 was considered statistically significant.

## Results

### Demographics and Clinical Features of XLH Patients

The study cohort included 153 patients belonging to 105 unrelated pedigrees: 87 were familial cases and 66 were sporadic cases. Of the 153 patients, 45 were male, and 108 female, with a median age and a median onset age of 23.0 years (range: 1.3–73.0 years) and 18.0 months (range: 9.0 months–26.0 years), respectively. The average height SDS for juveniles and adults was −2.3 ± 1.4 and −4.6 ± 2.1, respectively. According to further statistical analysis, adult patients showed significantly lower height SDS than juvenile patients (*P* < 0.001), which revealed a lasting effect on stature development during a long period of non-treatment. The most frequent manifestation of XLH in our center was bowed lower extremities, which accounted for 79.1% (121/153), followed by abnormal gait (106/153), short stature, growth retardation (106/153), dental disease (47/153), bone pain (28/153), and fracture (24/153) (Table 1). Lower-limb deformity or abnormal gait became apparent at a median age of 18.0 months. Based on radiographic manifestations, many patients had metaphyseal abnormalities of the distal femur, proximal tibia, and distal radius and ulna, with a mean RSS score of 4.8 ± 2.2 for juveniles (*n* = 34).

**TABLE 1:** Characteristics of 153 patients with XLH.

Clinical characteristics	All patients (*n* = 153)
Sex (Female: male), *n*	108: 45
Mean age, years	23.0
Mean onset age, months	18.0
Height (SDS^a^)	
Juveniles, mean ± SD	−2.3 ± 1.4
Adults, mean ± SD	−4.6 ± 2.1
Bowed lower extremities, *n*	121
Abnormal gait, *n*	106
Short stature, growth retardation, *n*	106
Dental disease, *n*	47
Bone pain, *n*	28
Fracture, *n*	24
Rachitic rosary, *n*	14
RSS^b^, mean ± SD	4.8 ± 2.2

*^a^SDS, standard deviation score. ^b^RSS, rickets severity score.*

### Biochemical Characteristics of XLH

Table 2 provides the biochemical characteristics of patients with XLH. Mean serum phosphorus was lower than the reference range established for all age groups (Ruppe, 1993). In addition, serum ALP values were greatly above the upper limit of normal in the juvenile group but were normal in the adult group. Furthermore, 54.4% of patients had serum PTH levels higher than the upper limit of normal, though serum calcium was within the normal range (*n* = 79), and 59.8% of patients had vitamin D deficiency (*n* = 82).

**TABLE 2:** Biochemical features of patients with XLH.

Biochemical parameters	Age groups	
Serum phosphorus, mmol/L	1–3 years	0.84 ± 0.20^a^
	4–11 years	0.79 (0.71–0.87)^b^
	12–15 years	0.75 ± 0.11
	>15 years	0.61 ± 0.11
Serum calcium, mmol/L		2.34 ± 0.12
ALP, U/L	1–15 years	585.5 ± 195.9
	16–18 years	388.7 ± 210.7
	>18 years	94.0 (82.5–146.0)
PTH, pg/ml		69.80 (56.39–86.28)
25OHD, ng/ml		17.84 (12.50–29.63)
iFGF23, pg/ml		91.88 (55.88–143.31)
β-CTX, ng/L		1,387.00 (491.00–2444.00)
OC, ng/ml		51.85 (21.38–99.55)
Serum creatinine, μmol/L		33.62 ± 13.62

*^a^Normally distributed data are shown as the mean ± SD. ^b^Non-normally distributed data are shown as the median (interquartile range). ALP, total alkaline phosphatase; PTH, parathyroid hormone; 25OHD, 25-hydroxy-vitamin D; β-CTX, β-CrossLaps of type 1 collagen containing cross-linked C-telopeptide; OC, serum osteocalcin in the form of an N-terminal mid-molecule fragment. Normal range: phosphorus [varies by age (Ruppe, 1993)]: 1–3 years: 1.25–2.10 mmol/L, 4–11 years: 1.20–1.80 mmol/L, 12–15 years: 0.95–1.75 mmol/L, >15 years: 0.80–1.60 mmol/L, calcium: 2.08–2.60 mmol/L, ALP: 1–15 years: 42–390 U/L (Zhang et al., 2019), 16–18 years: 52–171 U/L (Zhang et al., 2019), >18 years: 15–112 U/L, PTH: 15–65 pg/mL, 25OHD: >30 ng/ml, iFGF23: 16.1–42.2 pg/mL (Zhang et al., 2019), β-CTX: 278–540 ng/L (Hu et al., 2013), OC: 13.07–27.68 ng/mL (Hu et al., 2013), creatinine: 53.0–115.0 μmol/L.*

### Serum iFGF23 Measurement

Serum iFGF23 values were determined for 62 patients and displayed a wide variation from 14.39 to 730.70 pg/ml, with a median value of 91.88 pg/ml. For pediatric patients (*n* = 31), the median value of circulating iFGF23 was 94.87 pg/ml (interquartile range: 74.27–151.86 pg/ml), and 96.8% (30/31) of patients had a high level of serum iFGF23 (>42.2 pg/ml). For adult patients (*n* = 31), the median value of circulating iFGF23 was 72.82 pg/ml (interquartile range: 39.42–136.00 pg/ml), with 74.2% (23/31) having levels in the high range (Table 3). Despite the lack of difference in serum iFGF23 levels between these two groups (*P* = 0.062), the proportion of pediatric patients with high levels of circulating iFGF23 (>42.2 pg/ml) was higher than that of adult patients (*P* = 0.026).

**TABLE 3:** Distribution of serum iFGF23 levels in pediatric and adult patients with XLH (*n* = 62).

	Serum iFGF23 levels, pg/ml	No. (%)
Pediatric patients, *n* = 31	94.87 (74.27–151.86)	
	Low	0
	Medium	1 (3.2%)
	High	30 (96.8%)
Adult patients, *n* = 32	72.82 (39.42–136.00)	
	Low	1 (3.2%)
	Medium	7 (22.6%)
	High	23 (74.2%)

*Low: Serum iFGF23 levels < 16.1 pg/ml. Medium: Serum iFGF23 levels ≥ 16.1 and ≤42.2 pg/ml. High: Serum iFGF23 levels > 42.2 pg/ml.*

Correlation analysis demonstrated that serum iFGF23 levels had no relationship with the serum phosphate/upper limit ratio, age, onset age, height SDS or RSS (Table 4).

**TABLE 4:** Correlation analyses of circulating iFGF23 and other clinical and biochemical parameters.

Clinical and biochemical parameters	Serum iFGF23	
	*r*	*P* value
Age	−0.195	0.133
Age of onset	−0.077	0.590
Serum phosphate/Upper limit ratio	−0.195	0.159
Height SDS	0.259	0.064
RSS	−0.235	0.305

### Treatment and Following-up

Forty-seven (40.5%) of the 116 XLH patients who were treated with phosphate and calcitriol daily received follow-up; 28 were children, and 19 were adults, with a median follow-up duration of 12.0 months (3.0–120.0 months). For both groups, no significant increase in serum phosphorus level (*P* = 0.511; *P* = 0.651) or decrease in serum ALP (*P* = 0.434; *P* = 0.442) was observed at the median follow-up.

### Mutation Analysis of the *PHEX* Gene

A total of 88 different mutations were identified in 153 patients, including 15 missense mutations (17.0%), 15 non-sense mutations (17.0%), 21 splicing mutations (23.9%), 18 small deletions (20.5%), four small insertions (4.5%), and 15 gross deletions/duplications (17.0%). Of the 88 mutations, 27 (30.7%) were novel mutations not found in HGMD or reported in the literature. All the *PHEX* mutations identified in the present study are listed in Supplementary Table 1.

**FIGURE 1:**
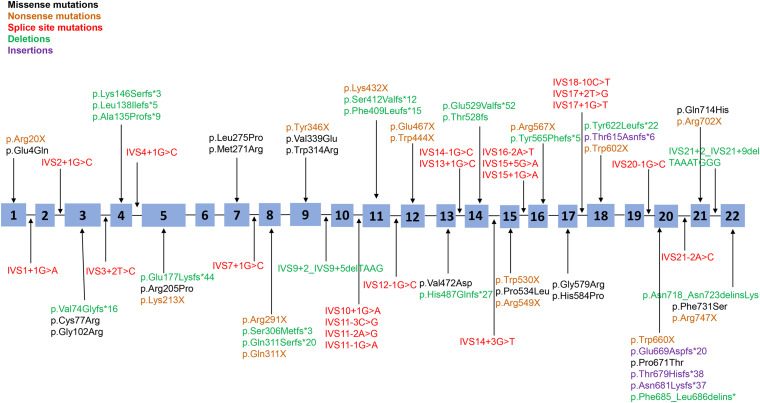
Distribution of all point mutations identified in this study. A total of 73 point mutations (including missense mutations, non-sense mutations, splice-site mutations, and small insertions/deletions) were scattered throughout the 22 exons and adjacent intron areas of the *PHEX* gene. Black corresponds to missense mutations, yellow corresponds to non-sense mutations, red corresponds to splice-site mutations, green corresponds to small deletions, and purple corresponds to small insertions.

Seventy-three point mutations, including missense mutations, non-sense mutations, splice-site mutations, and small insertions/deletions, are scattered throughout the *PHEX* coding sequence and flanking intronic sequences, with 80.8% of point mutations in the 5′ region (up to amino acid 649 in exon 19) and the other 19.2% in the 3′ region (amino acid 650 to the 3′ end of PHEX); no mutations were detected in the 3′-untranslated regions (UTR) or 5′-UTR (Figure 1). The most frequent point mutations were R702X and R549X, which accounted for 4.1% (3/73) and 4.1% (3/73) of all point mutations, respectively.

### Genotype-Phenotype Association Analysis

We divided the genotypes of the 153 patients into two groups, truncating mutations (non-sense mutations, deletions, insertions, and splice-site mutations, *n* = 124) and non-truncating mutations (missense mutations, *n* = 29), and explored the phenotypic severity of the groups. The results showed no significant difference in terms of the serum phosphate/upper limit ratio, serum ALP/upper limit ratio and iFGF23, age of onset, or RSS and height SDS. Because residues 1–649 of the PHEX protein include several functional domains, such as the transmembrane domain and the two zinc-binding motifs, mutations in these locations might result in a more severe phenotype (Holm et al., 2001). Therefore, we divided another two groups: N-terminal mutations (from 5′ end to amino acid residue 649, *n* = 105) and C-terminal mutations (from residue 650 to the 3′ end of PHEX, *n* = 24) to verify the above hypothesis. However, no significant difference in the serum phosphate/upper limit ratio, serum ALP/upper limit ratio, iFGF23, age of onset, RSS, or height SDS was observed between the two groups (Table 5).

**TABLE 5:** Genotype-phenotype correlation in patients with XLH.

	Truncating mutations (*n* = 124)	Non-truncating mutations (*n* = 29)	*P* value	N-terminal mutations (*n* = 105)	C-terminal mutations (*n* = 24)	*P* value
Onset age (years)	1.5 (1.0–2.0)	1.2 (1.0–3.0)	0.996	1.5 (1.0–2.0)	1.8 (1.2–3.0)	0.360
Height SDS	−3.5 ± 2.2	−3.9 ± 2.0	0.510	−3.5 ± 2.2	−3.4 ± 1.9	0.759
Serum phosphate/Upper limit ratio	0.40 ± 0.09	0.41 ± 0.07	0.925	0.40 ± 0.08	0.43 ± 0.12	0.286
Serum ALP/Upper limit ratio	1.29 ± 0.56	1.23 ± 0.42	0.700	1.36 ± 0.57	1.05 ± 0.36	0.077
Serum iFGF23 (pg/mL)	91.38 (60.96–141.99)	112.55 (40.74–337.53)	0.695	90.93 (53.55–142.08)	107.23 (72.07–160.31)	0.485
RSS	4.8 ± 2.2	5.0 ± 4.2	0.895	5.0 ± 2.2	4.4 ± 2.6	0.538

## Discussion

The present study describes the phenotypic characteristics and genotypic spectra of 153 pediatric and adult patients with XLH. Apparently, patients with XLH in China have similar symptoms to those of XLH patients in Western countries. Indeed, most patients present symptoms of rachitic skeletal deformities during childhood (Kinoshita and Fukumoto, 2018). In pediatric patients, their parents frequently discover lower-limb abnormities, or waddling gaits when they just learn to walk. A consensus statement of clinical practice for XLH (Haffner et al., 2019) indicates that rachitic skeletal characteristics usually occur at 6 months after birth and that waddling gait and lower-limb deformities become apparent at the age of 2 years. Correspondingly, our study showed that patients manifested bowed lower extremities or abnormal gait at a median age of 18.0 months.

Patients with XLH at our center exhibited typical biochemical traits, similar to those in Western countries. Ninety-five percent of these patients displayed decreased levels of serum phosphorus below the age-related reference range because of renal phosphate wasting, which is a result of increasing serum FGF23. Our study reported that 85.5% (53/62) of patients had serum iFGF23 levels above the upper limit of the reference range. However, there were still 12.9% (8/62) of patients having serum iFGF23 levels within the reference range, which suggested that serum iFGF23 in patients with XLH is very likely associated with other metabolic factors, such as serum PTH, circulating α-Klotho, hypoxia, and inflammatory cytokines, in addition to serum phosphate (Courbebaisse and Lanske, 2018). On the other hand, the serum concentration of iFGF23 is regulated by serum phosphate (Hori et al., 2016), serum FGF-23 is positively correlated with serum phosphate; hence, this “normal” FGF-23 in these patients can be considered inappropriately normal. It is worth mentioning that a 24-year-old woman who sought a medical consultation in 2014 had circulating iFGF23 levels under the reference range. We speculated the inappropriate level of iFGF23 in this patient might be due to the presence of hypophosphatemia; moreover, partial degradation of serum iFGF23 during long-term storage at −80°C may have resulted in the low level of iFGF23 for this patient. An *in vitro* study by Liu et al. (2003) illustrated that FGF23 is not the direct substrate cleaved by PHEX; in fact, the mechanism by which *PHEX* mutation causes overproduction of FGF23 remains unknown, and further study is required to clarify the pathogenesis.

To evaluate renal phosphate wasting, the tubular maximum reabsorption of phosphate per glomerular filtration rate (TmP/GFR) should be calculated clinically (Haffner et al., 2019). Unfortunately, we did not perform this, and further detection is needed to better evaluate the disease. Secondary hyperparathyroidism (SHPT), a typical property of XLH, was observed in 54.4% of patients prior to treatment in our center. Studies by DeLacey et al. (2019) and Rafaelsen et al. (2016) have also described the high prevalence of SHPT, at 83.3% (70/84) and 66.7% (10/15), respectively, in patients with XLH. Several factors may be associated with SPHT in patients with XLH. For those who receive treatment, prolonged oral phosphate induces intermittently elevated serum phosphate concentrations, with recurrent stimulation of the parathyroid glands (Mäkitie et al., 2003b); accordingly, calcitriol is required in combination with oral phosphate supplements in clinical practice to minimize the risks of SHPT. For those who have never received phosphate therapy, a reduced circulating level of 1,25(OH)_2_D due to excess FGF-23 is very likely the main factor involved in the development of hyperparathyroidism. In addition, loss of *PHEX* function in the parathyroid gland leading to abnormal PTH mRNA cleavage or degradation may be responsible for the occurrence of SPHT (Lecoq et al., 2020).

Until now, conventional therapy (phosphate salts and calcitriol) has been regarded as the first-line treatment option for patients with XLH in China. To assess the efficacy and safety of conventional therapy, serum ALP levels are suggested to be a reliable biomarker of rickets (Chesney et al., 1983; Tsuru et al., 1987; Linglart et al., 2014). In the present study, no improvement in serum ALP level was noted during the 1-year treatment follow-up. This may be explained by the notion that treating XLH with conventional therapy will increase circulating FGF23, which to some extent diminishes the therapeutic effect (Imel et al., 2010).

We identified *PHEX* gene mutations in this population-based cohort and found 88 different mutations in 153 patients. Of the 88 *PHEX* variants, 30.7% have never been reported, indicating that *PHEX* gene mutations are private (Rafaelsen et al., 2016).

Fifteen different missense mutations were identified in this study, of which six (40%) are novel. All missense mutations we detected occur at residues that are highly conserved in mammals. The mutation C77R is located at the site where five of the 10 conserved cysteine residues are clustered at the C-terminus of the transmembrane domain (Sabbagh et al., 2000). Consequently, such a mutation most likely disrupts disulfide bond formation and alters the secondary structure of the protein by influencing folding, leading to instability and dysfunction of the protein (Rowe et al., 1997). Additionally, G579R is very likely a mutational hot spot because approximately 20 unrelated patients of distinct races are reported to harbor this mutation (Popowska et al., 2000; Sabbagh et al., 2000, 2001, 2003; Durmaz et al., 2013; Radlović et al., 2014; Lin et al., 2018; Zhang et al., 2019). Our center detected the same mutation in a 3-year-old boy. G579 is adjacent to the highly conserved zinc-binding motif (HEFTH), a major fingerprint of the M13 family of metallopeptidases. Evidence-based research has demonstrated that the G579R mutation attenuates the function of the *PHEX* protein by altering protein trafficking, endopeptidase activity, and protein conformation (Sabbagh et al., 2003).

Non-sense and splicing site mutations are considered to cause truncation of the PHEX protein, resulting in loss of function. In the present study, the first N-terminal non-sense mutation was encountered in exon 1 (R20X), with this non-sense mutation segregating in two families. The R20X mutation is predicted to result in degradation of the mRNA through non-sense-mediated mRNA decay, thereby abrogating synthesis of a truncated protein (Sabbagh et al., 2000; Charoenngam et al., 2019). Another stop mutation in exon 21 (R702X), despite being located in the 3′ end of *PHEX*, has been proven to be deleterious. This mutant lacks two conserved cysteine residues, causing protein structure alteration and indicating that the end of N-terminal amino acids still has an important role in PHEX function. Among the 21 splicing site mutations in the current study, 12 are splice donor mutations, and nine are splice acceptor mutations. These splicing mutations result in major changes in the secondary structure of the PHEX protein *via* exon skipping, intron retention, and activation of cryptic splice sites (BinEssa et al., 2019).

The genotype-phenotype correlation of the *PHEX* gene mutation in XLH has not been well established, and scholars have followed genotype-phenotype correlation research with great interest, particularly regarding the relationship between phenotype and mutation type or location (Holm et al., 2001; Cho et al., 2005; Rafaelsen et al., 2016; Zhang et al., 2019; Zheng et al., 2020). An evidence-based study by Zheng et al. (2020) has revealed that the severity of the disease is not related to truncating or non-truncating mutations, as based on their experimental finding that truncating and non-truncating variants possess a similar functional portrait. Nevertheless, a study by Zhang et al. (2019) has concluded that patients with mutations in the 5′ region of *PHEX* have an earlier age of onset and higher circulating levels of iFGF23 than those with mutations in the 3′ region of the gene. Moreover, a study by Holm et al. (2001) has revealed no relationship between disease severity and the type or location of the mutation, despite a trend toward a more severe skeletal phenotype in familial patients with truncating mutations (*P* = 0.072). In conformity with the reports by Zheng et al. and Holm et al. no correlation between phenotype severity and truncating and non-truncating mutations or N-terminal and C-terminal mutations was found in our study.

In conclusion, our retrospective study broadens the genotypic spectrum of XLH. We have identified 88 different mutations in 153 Chinese patients with XLH, and 27 of the mutations have never been reported. Furthermore, the present study has determined circulating iFGF23 levels in XLH patients and found no relationship with age, onset age, severity of the disease or mutation type.

## Data Availability Statement

The datasets presented in this study can be found in online repositories. The names of the repository/repositories and accession number(s) can be found in the article/Supplementary Material.

## Ethics Statement

The studies involving human participants were reviewed and approved by Ethics Committee of Shanghai Jiao Tong University Affiliated Sixth People’s Hospital. Written informed consent to participate in this study was provided by the participants’ legal guardian/next of kin.

## Author Contributions

XL and SL conducted the study and analyzed the data. XL wrote the draft of the manuscript. ZZ and HY supervised the study and revised the manuscript. All authors read and approved the final manuscript.

## Conflict of Interest

The authors declare that the research was conducted in the absence of any commercial or financial relationships that could be construed as a potential conflict of interest.

## Footnotes

1 http://bioinfo.ut.ee/primer3-0.4.0/

2 http://droog.mbt.washington.edu/poly_get.html
