# Supplementary material for: Clinical and Genetic Characteristics of 153 Chinese Patients With X-Linked Hypophosphatemia
Source: Front Cell Dev Biol. 2021 Jun 1;9:617738. doi: 10.3389/fcell.2021.617738 (PMC8204109; doi:10.3389/fcell.2021.617738)
Supplement: Supplementary file 1 [file Table_1.DOCX]

**Supplemental Table 1. all the *PHEX* mutations identified in the cohort**

| **Point mutations** | | | |
| --- | --- | --- | --- |
| **EXON/INTRON** | **cDNA** | **Protein** | **Mutation type** |
| EXON 1 | c.10G>C | p.Glu4Gln | Missense mutation |
| EXON 1 | c.58C>T | p.Arg20X | Nonsense mutation |
| INTRON 1 | c.118+1G>A |  | Putative aberrant splicing |
| INTRON 2 | c.187+1G>C |  | Putative aberrant splicing |
| EXON 3 | c.221delT | p.Val74GlyfsX16 | Deletion |
| EXON 3 | c.229T>C | p.Cys77Arg | Missense mutation |
| EXON 3 | c.304G>A | p.Gly102Arg | Missense mutation |
| INTRON 3 | c.349+2T>C |  | Putative abberrant splicing |
| EXON 4 | c.402delA | p.Ala135ProfsX9 | Deletion |
| EXON 4 | c.411_414del | p.Leu138IlefsX5 | Deletion |
| EXON 4 | c.436_436+1delAG | p.Lys146SerfsX3 | Deletion |
| INTRON 4 | c.436+1G>C |  | Putative abberrant splicing |
| EXON 5 | c.528delT | p.Glu177LysfsX44 | Deletion |
| EXON5 | c.614G>C | p.Arg205Pro | Missense mutation |
| EXON 5 | c.637A>T | p.Lys213X | Nonsense mutation |
| EXON 7 | c.812T>G | p.Met271Arg | Missense mutation |
| EXON 7 | c.824T>C | p.Leu275Pro | Missense mutation |
| INTRON 7 | c.849+1G>C |  | Putative abberrant splicing |
| EXON 8 | c.871C>T | p.Arg291X | Nonsense mutation |
| EXON 8 | c.917delG | p.Ser306MetfsX3 | Deletion |
| EXON 8 | c.931C>T | p.Gln311X | Nonsense mutation |
| EXON 8 | c.931delC | p.Gln311SerfsX20 | Deletion |
| EXON 9 | c.940T>C | p.Trp314Arg | Missense mutation |
| EXON 9 | c.1016T>A | p.Val339Glu | Missense mutation |
| EXON 9 | c.1038C>A | p.Tyr346X | Nonsense mutation |
| INTRON 9 | c.1079+2_1079+5delTAAG |  | Putative aberrant splicing |
| INTRON 10 | c.1174-3C>G |  | Putative aberrant splicing |
| INTRON 10 | c.1174-2A>G |  | Putative aberrant splicing |
| INTRON 10 | c.1174-1G>A |  | Putative aberrant splicing |
| INTRON 10 | c.1173+1G>A |  | Putative aberrant splicing |
| EXON11 | c.1224delC | p.Phe409LeufsX15 | Deletion |
| EXON11 | c.1234delA | p.Ser412ValfsX12 | Deletion |
| EXON 11 | c.1294A>T | p.Lys432X | Nonsense mutation |
| INTRON 11 | c.1303-1G>C |  | Putative aberrant splicing |
| EXON 12 | c.1332G>A | p.Trp444X | Nonsense mutation |
| EXON 12 | c.1399G>T | p.Glu467X | Nonsense mutation |
| EXON 13 | c.1415T>A | p.Val472Asp | Missense mutation |
| EXON 13 | c.1461delT | p.His487GlnfsX27 | Deletion |
| INTRON 13 | c.1483-1G>C |  | Putative aberrant splicing |
| INTRON 13 | c.1482+1G>C |  | Putative aberrant splicing |
| EXON 14 | c.1584_1585+1delAG | p.Thr528fs | Deletion |
| EXON 14 | c.1586_1586+1delAG |  | Putative aberrant splicing |
| INTRON 14 | c.1586+3G>T |  | Putative aberrant splicing |
| EXON 15 | c.1589G>A | p.Trp530X | Nonsense mutation |
| EXON15 | c.1601C>T | p.Pro534Leu | Missense mutation |
| EXON 15 | c.1645C>T | p.Arg549X | Nonsense mutation |
| INTRON 15 | c.1646-2A>T |  | Putative aberrant splicing |
| INTRON 15 | c.1645+1G>A |  | Putative aberrant splicing |
| INTRON 15 | c.1645+5G>A |  | Putative aberrant splicing |
| EXON 16 | c.1694delA | p.Tyr565PhefsX5 | Deletion |
| EXON 16 | c.1699C>T | p.Arg567X | Nonsense mutation |
| EXON 17 | c.1735G>A | p.Gly579Arg | Missense mutation |
| EXON 17 | c.1751A>C | p.His584Pro | Missense mutation |
| INTRON 17 | c.1769-10C>T |  | Putative aberrant splicing |
| INTRON 17 | c.1768+1G>T |  | Putative aberrant splicing |
| INTRON 17 | c.1768+2T>G |  | Putative aberrant splicing |
| EXON 18 | c.1806G>A | p.Trp602X | Nonsense mutation |
| EXON 18 | c.1843dupA | p.Thr615AsnfsX6 | Insertion |
| EXON 18 | c.1863_1870del | p.Tyr622LeufsX22 | Deletion |
| INTRON 19 | c.1966-1G>C |  | Putative aberrant splicing |
| EXON 20 | c.1979G>A | p.Trp660X | Nonsense mutation |
| EXON 20 | c.2002_2006dup | p.Glu669AspfsX20 | Insertion |
| EXON 20 | c.2011C>A | p.Pro671Thr | Missense mutation |
| EXON 20 | c.2033dupT | p.Thr679HisfsX38 | Insertion |
| EXON 20 | c.2039_2042dup | p.Asn681LysfsX37 | Insertion |
| EXON 20 | c.2054_2057delTCCTinsA | p.Phe685_Leu686delinsX | Deletion |
| INTRON 20 | c.2071-2A>C |  | Putative aberrant splicing |
| EXON 21 | c.2104C>T | p.Arg702X | Nonsense mutation |
| EXON 21 | c.2142G>C | Gln714His | Missense mutation |
| INTRON 21 | c.2147+2_2147+9del |  | Putative aberrant splicing |
| EXON 22 | c.2154_2169delinsA | p.Asn718_Asn723delinsLys | Deletion |
| EXON 22 | c.2192T>C | p.Phe731Ser | Missense mutation |
| EXON 22 | c.2239C>T | p.Arg747X | Nonsense mutation |

| **Gross Deletions/Duplications** | | |
| --- | --- | --- |
| **EXON/INTRON** | **cDNA** | **Mutation type** |
| EXON 1-2 |  | Deletion |
| EXON 2 |  | Deletion |
| EXON1-3 |  | Deletion |
| EXON 6-11 |  | Duplication |
| EXON 8,16,20 |  | Duplication |
| EXON 10-12 |  | Duplication |
| EXON 12 |  | Deletion |
| EXON 12 |  | Duplication |
| EXON 15-20 |  | Deletion |
| EXON 15-22 |  | Deletion |
| EXON 16 |  | Deletion |
| EXON 16-22 |  | Deletion |
| EXON 19-20 |  | Deletion |
| EXON 21-22 |  | Deletion |
|  | 3324bp del; 34bp ins | Deletion; Insertion |
